# Expectations and Experiences of Couples Receiving Therapy Through Videoconferencing: A Qualitative Study

**DOI:** 10.3389/fpsyg.2019.02992

**Published:** 2020-01-21

**Authors:** Andrea Kysely, Brian Bishop, Robert Kane, Maryanne Cheng, Mia De Palma, Rosanna Rooney

**Affiliations:** School of Psychology, Faculty of Health Sciences, Curtin University, Perth, WA, Australia

**Keywords:** qualitative analysis, online therapy, videoconferencing, couples therapy, client expectations, client experiences, relationship education

## Abstract

Videoconferencing is an emerging medium through which psychological therapy, including relationship interventions for couples, can be delivered. Understanding clients’ expectations and experiences of receiving therapy through this medium is important for optimizing future delivery. This study used a qualitative methodology to explore the expectations and experiences of couples throughout the process of the Couple CARE program, which was delivered through videoconferencing. Fifteen couples participated in semi-structured interviews during the first and last sessions of the intervention. The interviews were conducted using the iChat program, with the therapist conducting the first interview and an external interviewer conducting the second. Thematic analysis was used to identify themes from the interview transcripts. Five themes were identified from the pre-therapy interviews, reflecting couples’ initial impressions and expectations: new experience, comparison, practical aspects, connection and dynamics, and distance and space. Couples’ experiences were explored in the eight themes from the post-therapy interviews: technicalities, the idea of “distance,” satisfaction and comfort, confidentiality, comparisons, new experience, expectations change, and working alliance. Overall, the present study found that couples experienced a positive shift in expectations. Despite some initial concerns regarding the therapist’s ability to empathize over a screen and the potential for the technology to break down, many clients noted that videoconferencing allowed them to become fully immersed in the therapeutic process. In fact, many couples felt that videoconferencing created an element of ‘distance’ from the therapist that allowed them to feel a greater sense of control and comfort. Couples consistently described being able to effectively connect with the therapist, and that the video actually enhanced the therapeutic alliance, due to a greater perceived focus on therapy processes. Overall, despite some initial concerns, the majority of couples found the videoconferencing experience to be beneficial and positive.

## Introduction

Rising rates of relationship distress and breakdown within Australia have led to increased demand for couple’s therapy; however, due to a scarcity of available services this demand is not consistently being addressed (Petch et al., 2014; Halford et al., 2015; Doss et al., 2017). While psychological therapies have historically been conducted face-to-face, the rising demand for accessible mental health services, coupled with the advanced development of modern technology has rendered online therapies an increasingly viable method of service provision (Castelnuovo et al., 2003; Simmons, 2006; Lungu and Sun, 2016). The advantages of health services delivered using technology are many, including lower cost, reduced stigma and increased access (Kruse et al., 2017). This technology is particularly valuable in Australia, where many individuals live in rural and remote areas with little access to services (Oakes et al., 2007; Richardson et al., 2009; Simpson and Reid, 2014).

Of the many online therapies available, videoconferencing is the most similar to real world interaction, as it enables both verbal and visual cues to be transmitted in real time (Castelnuovo et al., 2014; Deane et al., 2015).

Efficacy studies of videoconferencing have consistently reflected favorable outcomes in the areas of: client satisfaction, efficacy as compared to face-to-face therapy, clinical measures and the therapeutic alliance (Frueh et al., 2007; Richardson et al., 2009; Backhaus et al., 2012; Duncan et al., 2014). However, despite evidence of efficacy and increased awareness of this medium, few studies have examined the applicability of videoconferencing to couple’s therapy and the literature in this area is largely quantitative or theoretical (Backhaus et al., 2012; Doss et al., 2017; Wrape and McGinn, 2018). For this reason it remains essential to explore the lived experiences of those who have received couple’s therapy through videoconferencing (Elliott, 2008). Understanding the experiences of clients may inform the therapeutic process and provide a unique perspective on its outcomes.

### Expectations

A well-researched area in face-to-face therapy is client expectations, and furthermore the relationship between these expectations, attitudes toward counseling, and clients’ willingness to engage in and continue therapeutic intervention (Jacobson and Truax, 1991; Rochlen et al., 2004; Kimberly, 2005). Studies that have examined client expectations have linked these to attitudes, willingness to engage and utilize therapeutic services, and outcomes of the intervention (Constantine and Arorash, 2001; Beattie et al., 2009; Tambling and Johnson, 2010).

Given the comparative novelty of therapy presented using technological mediums such as videoconferencing, expectations of the process or the therapist may be inaccurate (Rochlen et al., 2004; Nguyen et al., 2012). Although videoconferencing shares many of the features of face-to-face therapy, the superficial distance between the therapist and client may influence clients’ motivation or willingness to engage (Suler, 2001). Educating clients about technological mediums as well as about psychological intervention in general, has been integral in creating more realistic expectations (Rochlen et al., 2004). It is of interest that research on the use of videoconferencing to deliver therapy for PTSD, for example, found that initial discomfort and negative perceptions did not impact overall on the development of a therapeutic alliance (Germain et al., 2010).

### Experience and Therapeutic Alliance

The study of client experiences is essential in understanding the implications on therapeutic processes, and subsequent outcomes (Elliott, 2008). In previous studies, clients have described their experiences of videoconferencing as more comfortable than traditional face-to-face counseling (Lewis et al., 2004). This may be due to the lack of physical presence of the counselor, allowing clients to feel less intimidated and awkward in the experience (Lewis et al., 2004). Some clients have also reported a deeper emotional experience than initially expected, allowing them to feel immersed in the counseling process. Meanwhile other clients have reported an increased sense of empowerment and control resulting from the sharing of a common space that belongs to neither the client, or the therapist (Lewis et al., 2004). Clients have also identified the advantage of being able to engage in therapy from their homes (Beattie et al., 2009).

However, responses to online therapy have not been consistently positive. While some clients felt that they could have a meaningful relationship with their therapist, other clients have indicated an absence of closeness that impacted their experience (Beattie et al., 2009). Beattie et al. (2009) also identified some clients who thought that online therapy could be beneficial, but not for them personally. These findings highlight the mixed responses that have been identified within the field of online therapy, and indicate a strong need for more research on the topic.

The therapeutic alliance is an essential part of psychological therapy, having a strong influence on outcomes (Asay and Lambert, 1999). One of the questions that has been raised in videoconferencing research is whether the therapeutic alliance can be established with online mediums to the same degree as in face-to-face therapy. Research has found that clients have thought it possible to develop a positive and good quality working relationship with their therapist through technology (Beattie et al., 2009). A review indicated that there is consistent evidence of the therapeutic alliance being successfully developed across a range of online mediums, although the effect of this alliance on the outcomes was inconclusive (Berger, 2015). Some studies have found no difference in outcomes between online and face-to-face interventions (Bouchard et al., 2004; Hanley and Reynolds, 2009; Stubbings et al., 2013). However, other studies have found better results with face-to-face therapy (Mallen et al., 2005). In a recent study, meta-analytic results indicated that the alliance formed through videoconferencing was inferior to that of face-to-face therapy; despite this, the outcomes were non-inferior (Norwood et al., 2017).

### Couples Therapy

Couples therapy is unique, in that it requires the therapist to work with and build a therapeutic alliance with both members of the couple together. Furthermore, research on couples therapy has found that when both members of the couple perceive the alliance as strong, therapy outcomes are increasingly positive (Symonds and Horvath, 2004).

There are a number of interventions for couples that have displayed efficacy when tested with face-to-face delivery (Wadsworth and Markman, 2012; Kanter and Schramm, 2017). However, due to the heightened complexity associated with conducting couples therapy, there are a number of barriers that may prevent couples in need from accessing this service. These can include geographical proximity and alternate schedules (Christensen et al., 2009; Farmer, 2009), as well as the stigma associated with accessing couples therapy, particularly in rural and sparsely populated areas where the general community may become aware of a couple entering therapy (Cicila et al., 2014). Many of these difficulties can be overcome through videoconferencing, allowing couples to overcome geographical barriers, and more discreetly and conveniently access psychological interventions (Pollock, 2006).

However, despite this growing need, few online interventions for couples exist, and this area remains under researched, with a scarcity of studies qualitatively exploring the experience of couples who have undergone online couples therapy, particularly via videoconferencing (Backhaus et al., 2012; Wrape and McGinn, 2018). This presents a significant gap in the literature, with a strong need for research directly exploring the unique dyadic relationship experienced by a couple when working with a therapist through videoconferencing. The present research is the first within Australia to use qualitative methods to focus on the experiences of couples using videoconferencing to engage with a relationship intervention. This area of research is particularly relevant in Australia given high rates of relationship distress and the geographical challenges that may hinder couples from accessing face-to-face therapy.

For these reasons, the current study used qualitative inquiry to investigate the expectations and experience of couples engaging in the Couple CARE program, a relationship intervention that has displayed efficacy when delivered face-to-face and over videoconferencing (Halford and Bodenmann, 2013; Kysely, 2015). This methodology will enable the researchers to evaluate the program on a number of levels and gain suggestions on how to improve the process.

This study aimed to use qualitative methodology to investigate and understand participant experiences of undertaking couple’s therapy–the Couple CARE program–through videoconferencing (Kysely, 2015).

The research questions explored in this study were:

1. What are couples’ initial impressions and expectations about engaging in couples therapy conducted via videoconferencing?
2. What were couples’ experiences of undertaking couples therapy conducted via videoconferencing?

## Methods

### Research Design

The present research was part of a wider study which implemented a mixed methods design, including both quantitative and qualitative components (Kysely, 2015). The quantitative component will be discussed in a separate article (Kysely, 2015). The current qualitative study took a phenomenological approach, using data from semi-structured interviews which were conducted at the beginning and end of the therapeutic process. In this way, participants’ expectations and lived experiences of couples therapy via videoconferencing were explored.

### Participants

Convenience sampling was used to recruit 33 couples through Curtin University, the wider community and local health services. Three couples dropped out during the study, leaving a total of 30 couples in the wider study. Fifteen of these couples received the Couple CARE program in a face-to-face condition and thus were not included in the thematic analysis for the present study. Therefore, the present study included data from 15 couples, or 30 individuals, who received the Couple CARE program via videoconferencing.

Inclusion criteria were met if both members of the couple were over the age of 18, were in a defined *de facto* or marital relationship, and were experiencing mild relationship distress assessed through interview and the Dyadic Adjustment Scale. Couples were excluded from the present study if they displayed any risk of suicidal ideation, were participating in a current couples therapy intervention, had a DSM-IV diagnosis of psychosis or schizophrenia, severe alcohol/substance dependence, concurrent psychological treatment, or clinically significant relationship distress. These criteria were assessed through an initial phone interview as well as through measures sent out as part of initial screening for participation.

The study aimed to recruit enough participants to achieve data saturation, the point at which any further information will add nothing new to the already collected information (Gehart et al., 2001). Based on previous research, which suggests that between 4 and 14 couples are needed to reach saturation, with an average of nine, the current study recruited 15 couples to the videoconferencing condition (Gehart et al., 2001). Participants were aged between 21 and 69 years, with a mean of 42 years. The length of couples’ relationships spanned between one and 49 years, with an overall mean of 10 years. Of the participants, 96.7% had completed high school and 50% had completed university. Eighty percent were currently employed, and 70% identified as Australian.

### Materials

Interview schedules were used to guide the semi-structured interviews conducted during the first and last sessions of the program. The questions were based on those used in similar previous studies (Krum-Heller Roe, 2002; Razzhavaikina, 2007). The pre-therapy interview schedule contained five questions, as well as prompts, which were used by the therapist. The post-therapy schedule contained 11 questions with prompts, which were used by an external interviewer. The content of both the pre-therapy and post-therapy interview schedules is displayed below (see Table 1). The ninth question from the post-therapy schedule was not needed in this study, as all couples were in the same room.

**TABLE 1:** Interview schedules.

Pre-therapy schedule	Post-therapy schedule
1. What are your initial impressions?	1. Have your expectations changed since beginning this therapy?
2. What do you expect from therapy in general?	2. Can you describe to me your experience of using videoconferencing for couple’s therapy?
3. Do you think that using videoconferencing will affect this in any way?	3. When you reflect on your experience, what stands out as meaningful? 4. Do you believe the camera affected the interaction between yourself and the therapist?
4. Do you expect any challenges?	5. Can you think of an example when it was easy for you to tell the therapist about something?
5. Are there any positive elements of this you are looking forward to?	6. Can you think of an example when it was hard to tell your therapist something? 7. Was there a time you wished you were in the same room as your therapist?
	8. Do you believe the camera affected the interaction between yourself and your partner?
	9. Was there a time when you wished you were in the same room as your partner? (if applicable)
	10. Can you describe your relationship with your therapist?
	11. Is there anything else you would like to tell me about your experience of couple’s therapy via videoconferencing?

The program was delivered to couples in the videoconferencing group using the iChat program on Apple Mac computers. This program allowed for the therapist and couple to see and hear each other in real time, and for the therapist to record all sessions, including the interview components. One computer was situated in the therapist’s office, while another was in a separate room for use by couples. The therapist organized the connection before each session began, allowing the session to begin when the couple entered the room. While the therapist was able to see both the couple and themselves, the couple could see only the therapist. This was done to prevent participant distraction (Simpson et al., 2005). NVivo9 software was used on a computer to assist with the thematic analysis.

### Procedure

Ethics approval for the present study was granted by the Curtin Research Ethics Committee (HREC No. # HR157/2011). After screening, couples who met inclusion criteria provided written informed consent and were randomized to receive the program either face-to-face or via videoconferencing. Couples attended their first session of the Couple CARE program at the Curtin University psychology clinic, either face-to-face or through iChat. During the first session, the therapist explained the study and any necessary information to the participants. The therapist and the couples also discussed their objectives and requirements for taking part in the program. The therapist then conducted the semi-structured interviews with participants.

The pre-therapy semi-structured interviews were conducted at the end of the first session, with both members of the couple participating in the interview together. The therapist asked each couple five open ended questions as outlined in the pre-therapy interview schedule. Couples in both conditions participated in the interviews to avoid the confound of unequal lengths of time spent with the therapist; the questions were modified for couples in the face-to-face condition. Each of these interviews lasted for approximately 15 min. The interviews were recorded and transcribed for analysis.

During the sixth and final session, couples were asked to participate in a semi-structured interview, where they had the opportunity to talk about their experience of receiving couples therapy through videoconferencing. Interviews were conducted by external interviewers who were undertaking postgraduate study in psychology. Interviews were conducted using the schedule provided (see Table 1). External interviewers were used to enable participants to speak freely about their personal experience of the therapist, without convoluting the therapist’s role. After the post-therapy interview, the qualitative stage of data collection was complete.

### Intervention

Couple CARE is a 6-week manualized intervention for couples that covers common areas of relationship functioning including self-change, communication, intimacy and caring, managing differences, sexuality and adapting to change (Halford et al., 2004). Each of these topics is explored in a separate week. The length of each session ranged from 50 to 60 min. The aim of the intervention is to strengthen relationships, increase satisfaction and functioning, and decrease distress. The program is educational and skills-based. Part of the intervention involves the therapist observing and discussing the interactions between members of the couple. The intervention aims to involve both members of the dyad to identify areas of behavior change and to assist them in changing these behaviors.

Sessions take place weekly for 6 weeks and couples are given tasks to complete between sessions. These tasks include watching a short video together each week followed by a number of weekly tasks to be completed individually and together between sessions. These tasks are then discussed and evaluated each week with a psychologist or counselor (Halford et al., 2004). Typically, this involves the therapist working together with the couple to devise a self-change plan, which includes tasks to enhance a different area of relationship functioning each week. The therapist then helps the couple to implement their plan and discuss any difficulties that may have arisen while completing their tasks. In this study, the therapist followed the manualized program and also gave the clients extra time to discuss anything else that they needed to speak about that week. She followed a set list of objectives for each session to maximize fidelity, for example, the objectives for the initial session (see Table 2).

**TABLE 2:** Example session objectives.

Objective
Introduction to program
Confidentiality
Provide feedback on questionnaires
Discuss program goals
Overview of unit
Discuss expectations of relationship
Discuss each partner’s expectations and set goals
Discuss vision of the relationship
Overview of self-change steps
Expectations about videoconferencing discussed
Set take-home exercises

### Analysis

The interview content was transcribed and analyzed to explore the lived experiences of couples who used the videoconferencing technology. Using an empirical form of phenomenology allowed for the subjective experiences of the couples to be explored in-depth in a unique and personal way (Finlay, 2009).

Thematic analysis was used to analyze the data, following the guidelines suggested by Braun and Clarke (2006). This was done to maintain rigor and validity throughout the process. After becoming familiar with the data, an initial list of codes was generated, and the data was searched for themes. As part of this process the transcribed interviews were entered into the computer program NVivo9 to clarify thematic groupings and create a thematic map. Following this, the identified themes were reviewed, named and defined. An external rater then re-analyzed the data to confirm the identified themes to ensure a rigorous analysis. Based on this, inter-rater reliability was calculated. The final phase involved writing up and reporting the findings.

## Findings and Discussion

The thematic analysis of the pre-therapy interview transcripts generated five main themes which reflected couples’ expectations and initial impressions of receiving therapy through videoconferencing (see Table 3). The post-therapy themes reflected the couples’ lived experiences of engaging in couples therapy as well as their perceptions of the therapeutic alliance.

**TABLE 3:** Pre-therapy themes.

Theme	Example quote
New experience	*I’m happy with it, it’s really clear, like I’m surprised at how clear it is to listen to you, and how the picture is as well, and how well it’s gone.*
Comparison	*When we did counseling before I remember it being quite emotional, you can see and feel the vibe in the room, it’s a bit less like this, ah which might keep more balance, controlled discussion if you like.*
Practical aspects	*As long as the audio is good, and the video is good, and it’s constant and there’s no hiccups. Like technology has a great tendency to let you down, the last thing I would want to be doing is pouring my heart out and then have that snap in the middle and have to stop and do it again, that would be terrible.*
Connection and dynamics	*Probably less daunting experience I mean to talk to a screen as opposed to a person. Not that I mind, just ah it’s um you feel close enough, but not too close.*
Distance and space	*It just seems a bit foreign and detached, so, and don’t get me wrong I’m not saying it’s not valuable, it’s very valuable, it could be so much more in that person attraction.*

### Pre-therapy Themes

The themes elicited from the pre-therapy interviews were: new experience, comparison, practical aspects, connection and dynamics, and distance and space.

#### New Experience

Couples reflected that videoconferencing was a new experience for them. There were varying responses stemming from this, with several clients noting that they had no expectations when entering the therapy, and some being skeptical about how effective it would be.

Some couples reported that this new experience was strange or “weird” as illustrated by the quote:

*“I find it really quite weird because there’s the door opened, there’s something about the lack of human interaction with it, it’s fine when you’re on the screen*…*I feel myself wanting a bit more softness around*…*but it’s just a bit institutionally awkward.” [4]*

Alternatively, other responses reflected that couples found the new experience acceptable and comfortable:

“I’m happy with it, it’s really clear, like I’m surprised at how clear it is to listen to you, and how the picture is as well, and how well it’s gone.” [20]

The expectations of couples at the beginning of the therapeutic experience can influence their willingness to engage in therapy, especially if it is a new experience for them (Caspar, 2005). As online therapy is relatively new, it is particularly important to examine client expectations, especially as they may not be accurate (Nguyen et al., 2012). Having an idea of common expectations can act as a guide for content to include in education about online therapy, so that clients are better prepared to engage in it.

#### Comparison

Couples tended to compare their experience to previous face-to-face interactions. Research indicates that clients who have engaged in face-to-face therapy previously, are likely to have formed expectations of online therapy due to prior experience (Suler, 2001). Some couples who expressed their preference for videoconferencing reflected that the reduced emotionality as a result of the technological medium would result in better outcomes due to more focus on content:

“When we did counseling before I remember it being quite emotional, you can see and feel the vibe in the room, it’s a bit less like this, ah which might keep more balance, controlled discussion if you like.” [21]

The finding that some couples saw reduced emotionality as being better was interesting, as some experts would consider this as a lack of rapport and alliance, resulting in less progress (Symonds and Horvath, 2004). However, some couples in this study indicated that this helped them to be more open in their disclosures, and more engaged in therapy. This is a recurring finding across studies, with clients commenting about being more empowered to speak about emotions over video, as opposed to face-to-face (Mitchell et al., 2003; Lewis et al., 2004; Simpson, 2009). This may be due to clients feeling less scrutinized or embarrassed when engaging in online mediums (Mitchell et al., 2003; Lewis et al., 2004).

In contrast to this, other couples saw videoconferencing as different to face-to-face therapy, and in some cases found it inferior:

*“Look I think it would, this is a good thing, I just think it would be better face to face. And don’t get me wrong*… *it’s not that I’m not used to VC, I just think I don’t know maybe being a people person*…*if I was to, just make a better connection I think.” [1]*

Other couples did not expect the technology to have any effect on the therapy, delivery of content, or relation to the therapist:

“I don’t think whether you’re here via the screen or sitting opposite us, it’s going to make any difference to how we relate to you and how seriously we take this, I don’t think that’s going to matter.” [14]

#### Practical Aspects

This theme reflected couples’ expectations of the practical aspects of videoconferencing. Several couples reflected on the location of where the therapy actually took place. Couples commented on the nature of the clinic but also expressed that they did not expect it to influence the therapeutic experience:

*“But the room is not conducive in terms of furniture*…*it’s sterile*…*it’s not going to affect us, it’s not going to affect us and the way we develop through the process.” [28]*

As a further reflection on location, couples suggested the possibility of engaging in therapy from their own homes. Some couples expected that they would feel more comfortable if they could engage in the process from their own homes, as illustrated by the quote:

“If it’s in your own home, again I think there’s huge benefit of you being in your comfort zone, and that could actually alleviate the other thing I talked about, the openness, cos I do feel like if you are in your own environment then you’re more relaxed, more comfortable about being as open as possible.” [2]

Couples commented about the physicality of the therapist and the way this was portrayed through the screen. There were a range of opinions on this topic, with some clients feeling more relaxed having the therapist on the screen:

“I know I feel a bit more relaxed in terms of body language, than if there was another person in the room.” [8]

Clients also discussed potential difficulties, such as the technology failing or people being unable to use it:

“As long as the audio is good, and the video is good, and it’s constant and there’s no hiccups. Like technology has a great tendency to let you down, the last thing I would want to be doing is pouring my heart out and then have that snap in the middle and have to stop and do it again, that would be terrible.” [28]

“With people who may not have access to IT it might be difficult, like if I gave this to your parents they just wouldn’t (use it).” [10]

Couples in this study held the expectation that high quality media was needed for a successful therapy experience. The literature on online therapy has also emphasized the importance of high standards of technology (LeRouge et al., 2002). Visual data is of particular importance, as it influences the therapist’s physicality, which in turn contributes to the clients’ experience (Sue and Sue, 2003). Certain characteristics, such as high image and sound quality, can enhance the perceived presence of the therapist in the room (Lombard and Ditton, 1997).

#### Connection and Dynamics

This theme explored the couples’ expectations about their ability to feel therapeutically connected to the therapist, as well as the resulting interpersonal dynamic resulting from this connection. Couples spoke about their initial impressions of connecting online:

“I found that we got acquainted very quickly you know, ah we probably got more done than you would have, like from my experience of a first meeting of someone.” [7]

This quote indicates that some couples felt the connection developed faster online, than it would be in a face to face setting. However, another couple reflected on initial feelings of doubt as to whether the therapist would be truly empathetic on the other side of the screen:

“As a client knowing whether or not there’s going to be real empathy on the other side of the screen, you know, that’s, and how that’s going to um flow, that’s just an interesting question on my mind at the moment.” [27]

This is a valid concern; however, research has found that it is in fact possible for emotions to be successfully conveyed through a technological medium (Bischoff, 2004).

Couples also spoke about the dynamics that were created between the three people involved, noting that videoconferencing created a more comfortable and less frightening space:

“Probably less daunting experience I mean to talk to a screen as opposed to a person. Not that I mind, just ah it’s um you feel close enough, but not too close.” [15]

Most clients had positive expectations about their connection with the therapist, and found that the dynamics of an online connection created a less threatening space. It is of note that clients expressed being more comfortable connecting through a screen than in person, as they had their own personal space. This has been a recurring theme in previous research (Lewis et al., 2004; Simpson, 2009). This indicates that the technological medium could be an alternative for couples who are not comfortable going to see a therapist face to face, for a number of reasons, such as anxiety or a fear of negative appraisal (Teyber, 2006; Christensen et al., 2009).

Concerns were raised about the potential for a volatile situation to become dangerous, without the physical presence of the therapist:

“It could be challenging if there was a violent relationship I guess, you know two people in a locked room with a therapist out there not being able to um yeh intervene in that instance.” [26]

Although couples in the study did not have safety concerns personally, this could be a very justified fear for other couples, which could lead to negative expectations of online couples therapy. This highlights the importance of having safety procedures in place, which couples are notified of prior to beginning therapy (Fenichel et al., 2002). Therapists are able to access best practice guidelines to assist them with implementing these safeguards (Wrape and McGinn, 2018).

#### Distance and Space

This theme explored the concept of a perceived distance that was created as a result of sharing a space over a technological medium. Some clients noted that the distance made them feel detached:

“It just seems a bit foreign and detached, so, and don’t get me wrong I’m not saying it’s not valuable, it’s very valuable, it could be so much more in that person attraction.” [1]

In contrast, other clients found that the distance allowed them to feel more open and comfortable:

*“In fact I think it is probably easier for someone like me*… *because um there’s like a third dimension to it in a way, so, but you still get the same contact, you don’t feel like you’re talking to a screen, you feel like there is a person there, but at the same time you’re kind of like in your own space, so you’re actually kind of well protected.” [24]*

This heightened comfort from being in a different physical space to the therapist can be linked to a phenomenon known as the online disinhibition effect. The online disinhibition effect refers to the phenomenon where individuals disclose information to a greater extent online, as compared to face-to-face (Suler, 2001). It has been suggested that self-disclosure in online therapy is more consistent than in face-to-face sessions, as people feel freer to express themselves in this context (Iacovelli and Johnson, 2012; Nguyen et al., 2012), and this idea was reflected in the present study.

### Post-therapy Themes

The broad themes elicited from the post-therapy interviews were: technicalities, the idea of ‘distance’, satisfaction and comfort, confidentiality, comparisons, new experience, expectations change, and working alliance (see Table 4).

**TABLE 4:** Post-therapy themes.

Theme	Example quote
Technicalities	*I think that if um sometimes*… *when it cuts out the little bit, sometimes that can make, you suddenly like, makes you feel like*… *there is like that distance between you.*
The idea of “distance”	*I think actually the video screen is good because you care, you care about what you’re going to say and um you there’s something, I’m not sure maybe to do with being one step removed, you know, so maybe there is a high level of responsibility*… *I felt like I thought carefully about what I was doing.*
Satisfaction and comfort	*I think it’s a lot easier*… *I think it’s a lot more comfortable than what we expected, because it is quite intimidating to think you are going to come here and talk to somebody, somebody that you don’t know, it is quite nerve racking.*
Confidentiality	*What could be a real issue*… *not knowing whose hands it could end up in, because yeah that would be a concern to me if I was a very private person, where this might end up.*
Comparisons	*I think that the experience has been very positive and the fact that it has been delivered via videoconferencing it’s, it’s even better in some aspects*… *and I don’t feel the sessions have been impaired at all via the means of communication.*
New experience	*I’ve never done any of it before so it was a whole new experience for me.*
Expectations change	*Surprisingly really helpful, it exceeded my expectations.*
Working alliance	*I thought her listening was amazing*… *she would come back with something that we said*… *she was obviously listening well*… *and she remembers things*…*her following up was amazing.*

#### Technicalities

Couples chose to reflect on certain physical characteristics, including the physical location and technological aspect involved in their videoconferencing experience. For example, one client noted that the simple act of the therapist not having to write notes due to the recording of sessions affected their experience:

“*I think that’s a positive aspect*…*that people don’t have to be taking notes you know from your side of the table, if you like, you know I think that’s very positive.*” *[27]*

Specifically, to this idea, another client added:

“*I hated hearing the pen, that just really unnerved me, and you couldn’t hear that because it was through the camera, so it made me feel more comfortable.*” *[9]*

This demonstrates how videoconferencing can in fact enhance the therapeutic experience. Furthermore, couples reflected again on the ‘technicalities’ of the therapist not being in the room with the couple:

“*there was a time where we needed a piece of paper, and she forgot to put it in the room, so then she needed*… *for somebody else to pick up and put it in here, so I think that was just*…*a small thing really.*” *[9]*

Again, this illustrates some of the real-world challenges of using videoconferencing that need to be considered in future endeavors. Some of these difficulties included the quality of the audio and visual information. Having clear and synchronized audio and video is essential to the quality of client care (Bouchard et al., 2000; LeRouge et al., 2002; Simpson and Reid, 2014). Some couples commented that at times the audio did cut out:

“*I think that if um sometimes*…*when it cuts out the little bit, sometimes that can make, you suddenly like, makes you feel like*…*there is like that distance between you*.” *[6]*

Missing words can reflect a potential fracture in the alliance as illustrated in the above quote, or even a subpar service delivery. Couples also reflected on the effect of the camera on their therapeutic experience. As the comment below illustrates, couples adjusted to the videoconferencing quickly and many subsequently stopped noticing that the therapist was not physically in the room:

“*I think that you forget that you’re actually*…*on camera, I think that I do.” [20]*

This participant’s quote reflects the idea of social presence, particularly the camera’s ability to convey the physical presence of the therapist to the couple, and vice versa (Rice, 1992; Holmes and Foster, 2012). More specifically, Muhlbach and Ptussong (1995) identified this idea of telepresence in videoconferencing, as the extent to which individuals in different locations, feel connected in one place. Others noted the camera, but did not perceive it to be a barrier to the therapeutic process:

“*I just pretended it wasn’t there, it was us talking, and it was ‘A’ on the other side, but you know we were talking, communicating.*” *[11]*

Meanwhile, other couples found the opposite, reflecting that there was an effect of the camera. Some couples consistently noted a distinct lack of intimacy conveyed through the screen:

“*yeah I think I would prefer to be in the same room as the therapist*…*I think the emotional content may be a little lost sometimes, across the screen*… *I still think that if something became really intimate*… *where there might be tears and such*…*I just think that there is a little bit of distance with some of those issues that might come up.*” *[27]*

Research suggests eye-contact, topic of conversation, facial and overall bodily expressions, all contribute to the amount of intimacy felt between two people (Vertegaal et al., 2003; Simpson et al., 2005). These same elements apply to individuals connected via a videoconferencing screen, as reflected in this theme.

#### The Idea of Distance

Couples also noted that videoconferencing created a form of “distance” between the couple and the therapist. Couples noted a level of comfort with the perceived distance, noting that this gave them more control as well as responsibility for the sessions. Furthermore, this responsibility couples saw for the therapeutic process of sessions, increased their focus and ensured they were both involved and exerting effort into making the sessions worthwhile for themselves:

“*I think actually the video screen is good because you care, you care about what you’re going to say and um you there’s something, I’m not sure maybe something to do with being one step removed, you know, so maybe there is a high level of responsibility*… *I felt like I thought carefully about what I was doing.*” *[2]*

As reflected in the pre-therapy theme “distance and space,” some couples also felt that the technology gave them a sense of having their own space and allowing them to feel more enabled by the technology. Feeling ownership of a space can empower a client to feel more in charge of the situation and can therefore allow the client to open up more as a result of feeling safe (Lewis et al., 2004). One couple reflected on this idea in particular of feeling safe in their own space:

“*a little bit of a safety zone I suppose because they’re over there, and your over here so, it’s actually quite a safe environment to actually be honest, and you’re sitting in a room so I can kind of say what I want to say, and you don’t really have that person’s energy, like reacting, you only have them yeah sort of visually reacting there, so that’s probably a good thing.*” *[24]*

However, other couples found the distance created by videoconferencing to be negative:

“*the impersonality of the videoconferencing as method of delivery is at first quite a barrier*… *it affects the relationship because they’re barriers, and in terms of the technology, um, quite hard edged, for a soft subject.*” *[4]*

This highlights that while many couples found the distance created by videoconferencing to be positive, some couples felt that it created a sense of impersonality within the therapeutic experience.

#### Satisfaction and Comfort

Many couples noted that they found the videoconferencing experience more comfortable and less threatening due to the lack of the physicality of the therapist. This finding is also consistent with those of previous studies (Lewis et al., 2004). A number of couples reflected on their experience of therapy via videoconferencing as facilitating discussion and communication that felt comfortable and relaxed:

“*I think it’s a lot easier*…*I think it’s a lot more comfortable than what we expected, because it is quite intimidating to think you are going to come here and talk to somebody, somebody that you don’t know, it is quite nerve racking.*” *[4]*

Couples also reflected on their satisfaction with the videoconferencing element of their experience:

“*it has been an enjoyable experience*… *as I say we’ve learnt a lot about ourselves and each other.*” *[12]*

It allowed for them to enter and engage with the therapeutic process despite not having the therapist physically present in the room:

“*there’s for you lot more positive coming out of videoconferencing counseling session then you would do if you were face-to-face with a counselor*… *you’re feeling extra comfortable, you’re not feeling like they’re intimidating because they are not in the same room as you, and they’re*…*sort of looking in and going ‘well why don’t you think of this, or think of this’ and then letting you two actually get to the problem.*” *[10]*

This couple reflected on the experience of being more comfortable as a result of videoconferencing, as well as feeling less intimidated or judged. Similar to findings by Callahan and Inckle (2012), the idea of feeling more in control over the session is very important as it empowers clients, and more specifically to the current study, allowed couples to feel they are benefitting from the session and feel confident enough to embrace the skills learnt independently.

#### Confidentiality

One of the most important ethical principles of therapy is confidentiality, this is especially poignant in the online therapies literature, due to the unique challenges afforded to such approaches (LeRouge et al., 2002; Deane et al., 2015; Gamble et al., 2015). In line with this, couples did note some concerns around confidentiality:

“*what could be a real issue*… *not knowing whose hands it could end up in, because yeah that would be a concern to me if I was a very private person, where this might end up.*” *[20]*

Couples then speculated that perhaps in such circumstances the videoconferencing could ultimately hinder the therapeutic process, due to the individual feeling restricted in what they felt comfortable to discuss and disclose:

“*maybe it could inhibit what I choose to talk about, and perhaps has, and perhaps haven’t gone into some areas that um that I might not want to talk about*…*on camera*.” *[20]*

For the most part couples reflecting on their own experience of videoconferencing in the current study did not feel that confidentiality had been a significant issue, however, as one couple noted, they felt prior to continuing the therapy they needed to ensure what they were saying could not be heard out of the room:

“*that after I left here, I’d hate to think that somebody’s been out there listening because I’m very insecure about my, about our business.*” *[14]*

LeRouge et al. (2002) noted that a key attribute of quality telemedicine is the provision of privacy and specifically quiet and soundproof locations for clients. Once couples were assured this was the case, they noted feeling more at ease to able to engage in discussion of personal topics.

#### Comparisons

Couples compared the therapy via videoconferencing to face-to-face interactions, with varied responses. Some found videoconferencing to be comparable to face-to-face interactions, while other couples expressed a preference for face-to-face interactions. One couple suggested that the videoconferencing is able to effectively simulate face-to-face to interactions, stating:

“*it’s real you know*…*because like you can see reactions and things, like it feels like they’re listening*…*the video makes it good.*” *[9]*

Some couples went a step further and noted that in addition to the videoconferencing experience being comparable to that of a face-to-face interaction, they found the medium to actually be preferable and superior:

“*I think that the experience has been very positive and the fact that it has been delivered via videoconferencing it’s, it’s even better in some aspects*…*and I don’t feel the sessions have been impaired at all via the means of communication.*” *[15]*

This quote reflects a consistent satisfaction with videoconferencing, however, this sentiment was not reflected by all couples, with some couples expressing a preference for face-to-face interaction:

“*I think I would prefer to be in the same room as the therapist*… *I think it’s fine either way, I think the emotional content may be a little lost sometimes, across the screen.*” *[27]*

In fact, some couples expressed a desire for a combined delivery method of both videoconferencing and face-to-face interaction:

“*it would have been great if we have the first appointment in person*…*I can see that working quite well.*” *[2]*

Another participant suggested:

“*Maybe if the service was offered once every 6 months to have met your therapist face-to-face*…*that could be an option.*” *[20]*

Overall, many couples reflected a consistent satisfaction with this medium when compared to face-to-face therapy, however, there remained some couples that preferred the physical interaction, especially in a therapeutic context.

#### New Experience

Couples also reflected on therapy through videoconferencing as a completely new experience, however, despite this many reflected on it as a positive experience:

“*came in with a very open mind um and I found it to be quite a good thing.” [23]*

Couples noted that it was a totally new experience for them:

“*I’ve never done any of it before so it was a whole new experience for me.*” *[25]*

However, despite it being an experience unlike any other they had had before, they decided to keep an open mind regarding use of the videoconferencing for the facilitation of the therapeutic process:

“*you’re in here for reason, and the other person on the other side of the screen is putting some input, making an effort, so keep an open mind and just see where it takes you.*” *[1]*

As reflected by these clients, many of the couples who noted that they had actually entered the program with an open mind found that they were satisfied with their consequent experience.

#### Expectations Change

Couples discussed how their expectations had changed since beginning the therapy. Here couples identified predominantly that their expectations had changed, largely for the better, often noting their initial expectations had actually been exceeded:

“*initially I wasn’t sure how effective it would be because of the screen thing, yeah so my expectations definitely changed for the better.” [1]*

This quote illustrates that some couples entered therapy somewhat skeptical about videoconferencing as a viable therapeutic tool. Similar to those clients in the Lewis et al. (2004) study, clients noted that once they had the opportunity to familiarize themselves with the technological medium of videoconferencing, they noted the experience being better than initially expected:

“*surprisingly really helpful, it exceeded my expectations.*” *[1]*

Another couple reflected that their ability to align themselves with the therapist and immerse themselves in the therapeutic process was not inhibited by the use of videoconferencing:

“*I didn’t know how it would work*…*I was really probably skeptical, how it would go*… *because you didn’t have that contact, that sort of face-to-face*…*now I think it’s probably, probably positive in terms of like I said I haven’t tried to prove to get the person on side*…*that’s way exceeded my expectations.*” *[19]*

Whilst this theme reflects this initial skepticism from couples regarding the videoconferencing medium, all comments are followed by the idea that after adjusting to the experience, in general it exceeded their expectations.

#### Working Alliance

Many couples reflected on their relationship with the therapist to a large extent. Symonds and Horvath (2004) performed research into how an alliance is established, maintained and most importantly perceived by a couple dyad prior to, and during couples therapy. They found that when the couple perceives the alliance as strong, and both partners agree on this strength, the positive outcomes of therapy are significantly greater (Symonds and Horvath, 2004). This idea is also reflected in the current study:

“*I think the whole of the way that it worked just made it that much better, like with them (the therapist) being separate, and being via video just made it much more comfortable, I think it built the relationship between us and her much better.*” *[10]*

However, as seen in the following quote, not all couples felt they could overcome, or adjust to the technological element of the video:

“*I still think that if something became really intimate*… *where there might be tears in such, and I know that it’s not the therapist’s role to cry along with you*…*empathize with the experience*…*I just think that there is a little bit of distance with some of those issues that might come up*…*using this medium.*” *[27]*

Couples also noted that participating in therapy via videoconferencing can potentially skew the power balance, and enhance the distance between the two:

“*in the first few sessions*…*in a way I guess when we’re talking, we’re talking to each other, and I could see out of the corner of my eye*…*’A’ trying to stop maybe you from continuing, or trying to point something out and that not coming across*… *(but) I think as it went on we probably became a bit more aware of it, and she may have been a bit more vocal about it.*” *[8]*

A number of couples noted that the dynamic actually enhanced their interaction with the therapist, and especially the other partner:

“*because you know there’s the two of us in this room*…*we’re the ones doing it, you know ‘A’ is more kind of guiding us as opposed to if she was in the room we might feel a little bit more like*…*we couldn’t do it on our own*… *it kind of makes it feel like it’s more easy for us to translate this into home.*” *[8]*

This quote indicates that the participants were able to take more ownership over the process as a couple, rather than feeling that they could only succeed if the therapist was physically there with them. This is encouraging, as it suggests that the online medium helped the members of the couple to more readily apply what they learnt in their own contexts, even after leaving the therapeutic setting. Many couples also described the experience of watching the interaction between one partner and the therapist more carefully, and thus being able to facilitate therapeutic progress more effectively:

“*you’re actually watching and listening and looking at the same time*… *and you know that ‘A’ is not reacting to me, or participating with me, she is participating with B. and when she participates with both of us then obviously it’s a three-way talk, so I felt that was a really good thing.*” *[24]*

The working alliance literature asserts that as therapy continues, a stronger relationship can develop between a therapist and client which can ultimately facilitate behavioral change and essentially enhance the therapeutic intervention (Asay and Lambert, 1999; Ardito and Rabellino, 2011). As seen below, couples were satisfied with the therapeutic alliance enabled through this medium:

“*I thought her listening was amazing*…*she would come back with something that we said*…*she was obviously listening well*…*and she remembers things*…*her following up was amazing.*” *[22]*

Couples also noted that the therapist allowed them to feel at ease to discuss sensitive topics:

“*well the sexual intimacy all went smoothly, the way it was handled and you know, um, I said to ‘A’ not something I normally talk about, but yet she made it all very easy, and it didn’t feel uncomfortable at all so it was well done.*” *[26]*

The need for a therapist to remain impartial and non-judgmental is critical in facilitating the establishment of a strong therapeutic alliance with a client (Symonds and Horvath, 2004), and this is reflected in the following quote:

“*I certainly felt she was quite you know impartial, or non-judgmental, so that was quite easy to sort of talk about anything*…*she would kind of guide you away from pointing out someone else’s problems*…*and guide you back into talking about yourself*…*I thought that was quite good.*” *[7]*

However, some couples noted that the ability for non-verbal behaviors to be conveyed through the screen, such as facial expression was inhibited somewhat by the medium:

“*your facial expressions, you pick up on more when somebody is in the room with you, and you notice the way they are looking as well. like if they are looking directly at you or them*…*but on the other hand like I still felt like the relationship with us and her was really good.*” *[6]*

Despite this, most couples described this not having a significant influence on their disclosures or engagement in the therapy.

## Discussion

Clients reflected on videoconferencing being a *new experience* both before and after therapy. Some clients found the experience “weird” at first, while others were comfortable with it from the beginning, or had minimal expectations. After participating in the program, the main idea in the new experience theme was that of clients coming in with an open mind, and subsequently having a satisfying experience. Similarly, the *comparison* theme was present both before and after. Before therapy, the idea was raised that videoconferencing was similar enough to face-to-face that it would not have an effect on the therapy. Some clients expected it to be better and some expected it to be worse. Interestingly, these ideas did not change after therapy. There were still a range of opinions, with preferences being expressed either way. Similar to previous studies, clients found that once they became immersed in the therapy itself, the technology became less of a focus (Mallen et al., 2005). This has been a recurring theme in online therapy literature, providing strong evidence that the experience of receiving therapy through videoconferencing is not subpar to that of face-to-face therapy, but instead “different” (Day and Schneider, 2002; Lewis et al., 2004; Simpson et al., 2005; Richardson et al., 2015).

*Practical aspects* were also discussed at both times. In the pre-therapy interviews, clients listed as potential distractors the characteristics about the room and physicality of the therapist, but did not expect that these would affect the process. An expectation raised was that the media quality would be high, and that this would contribute to the effectiveness of the process. After therapy, clients again referred to *technicalities*, such as audio cutting out, reflecting the real world challenges of using technology. However, many clients also expressed that after getting used to it, they stopped noticing that they were talking through a screen. Thus, it appeared that social presence was able to be transmitted over the online medium (Muhlbach and Ptussong, 1995). Another practical aspect discussed post-therapy was *confidentiality*, with couples speaking of the importance of what they said staying in the room. Some couples also reflected on the importance of having safety procedures in place, since the therapist was not in the same physical space as the couple.

Before therapy, clients had a range of expectations about the *connection and dynamics* between them and the therapist. Some felt that they were able to connect faster than in real life, while others were skeptical about the ability of the therapist to empathize online. Another theme related to the *distance and space* between the therapist and couple that was created by the use of videoconferencing. This distance made some clients feel detached, while others found that it made them feel more comfortable, as they had their own safe space. This finding highlights that videoconferencing could be an option for clients who feel uncomfortable meeting a therapist in a physical space (Christensen et al., 2009). After therapy, clients discussed the idea of having their own safe space as a result of being in a different physical place to the therapist. Some clients noted that they felt less threatened.

After therapy, most clients felt that they had been able to connect to the therapist and form a *working alliance*, and that in some cases, the online distance had enhanced this. This was similar to the findings of other studies, which have found that clients felt they could disclose more over online mediums (Lewis et al., 2004). Clients spoke about the three-way dynamic that was created as a result of using videoconferencing. This had a number of benefits, such as being able to observe their partner interacting with the therapist, and being able to take ownership of their relationship change as a couple, due to the therapist appearing to be a little more removed from the situation. Similarly, clients discussed *the idea of distance*, reflecting that it helped them to feel more in control and responsible for progress in the sessions. However, other clients felt that the distance made the experience seem impersonal and that, especially in more emotional situations, the distance would be an issue.

After completing the program, most clients reported a sense of *satisfaction and comfort* with using videoconferencing. This was especially apparent for clients who reported that they had tried to keep an open mind going into the program. Another theme was *expectations change*, with many clients noticing that their expectations had changed for the better throughout the process, which aligns with previous research findings (Asay and Lambert, 1999; Rochlen et al., 2004). Interestingly, a number of clients reflected on feeling more safe and comfortable using videoconferencing than they would have been face-to-face.

The findings of this study are particularly relevant in light of the Telehealth Medicare Benefits Scheme that currently exists in Australia (Australian Government Department of Human Services, 2019). This scheme provides help for clients who need to access health services but are facing barriers to doing so, such as distance or mobility. New items were introduced to the scheme toward the end of 2019, to promote the use of telehealth for clients living in rural and remote areas (Australian Government Department of Health, 2019). As the findings of this study indicate that clients found this program beneficial to them in a number of ways, it could potentially be taken up by mental health practitioners who need to deliver couples therapy online. There are few exiting couples therapy programs that have been tested using videoconferencing, so this program is unique in that respect.

### Strengths and Limitations

The present study is strengthened by the inclusion of a range of couples with various presenting issues, which increases the generalizability of the study to real world populations. The interview data came from a wide spectrum of couples, who differed in age, length of relationship and other characteristics. There were also enough couples to gather a range of differing responses.

However, due to limited resources and budget, the pre-therapy interviews were conducted by the same therapist who was going to deliver the remaining sessions of the program, presenting a limitation within the present study. Some quotes may have reflected that the clients did not want to offend the therapist, for example, *“and don’t get me wrong, I’m not saying it’s not valuable, it’s very valuable.”* If possible, in future studies, it may be better to have an external interviewer conduct these initial interviews. The therapist also conducted the analysis; however, an external reviewer was also asked to confirm the themes, to reduce the risk of bias.

### Future Research

Expectations of therapists, as well as those of clients, may have the potential to impact on the therapeutic process. Therapists have generally been more hesitant about delivering online therapy than clients are to receive it (Cowain, 2001). Research has found that therapists have tended to view online therapy as less effective than face-to-face therapy, which could influence the outcomes produced. Some therapists have also expressed concerns about the ability to convey warmth and empathy through videoconferencing (Rees and Stone, 2005). Future research could explore the expectations of therapists toward delivering couples therapy online.

Some couples raised the idea that therapy delivered through videoconferencing was better for them, as they might have been too shy, and therefore hesitant to engage in therapy face-to-face. Future research could investigate the effect of personality and other individual factors on clients’ expectations, and willingness to engage in online therapy. Online therapy may therefore be particularly beneficial for certain individuals or groups within the population who might not have been willing to engage in therapy in a face-to-face context (Christensen et al., 2009).

As suggested in the pre-therapy theme “practical aspects,” a future study could involve the couples participating in therapy from their own homes. This might generate a range of different expectations, due to the different physical context. Other variations to the study could include having both partners separate from each other, as well as clients accessing online therapy through different forms of technology, such as a mobile phone or tablet.

Another potential avenue to investigate is the efficacy of combined delivery methods, where videoconference could be delivered in combination with face-to-face therapy. This mode of combined delivery was requested by some study participants, as seen in the following quote:

*“Maybe if the service was offered once every 6 months to have met your therapist face-to-face*…*that could be an option.”*

Given some participants felt a preference to discuss highly emotive topics in a face-to-face setting, a combined delivery method may facilitate this, while still maintaining the benefits of videoconferencing.

## Conclusion

The present study aimed to explore the expectations and experiences of couples engaging in therapy through videoconferencing. At the beginning of the therapeutic process, couples reflected on their initial impressions and expectations, with many couples comparing their initial experience of videoconferencing against either previous therapy, or general face-to-face communication. The results reflect that overall, couples experienced a positive shift in expectations, with many couples’ initial reservations overtaken by their ability to become fully immersed in the therapeutic process. Whilst couples described the videoconferencing experience as “different,” it was not necessarily perceived to be worse, or more difficult. In fact, many couples felt that the distance created by the technology allowed to feel a greater sense of control and comfort.

Couples were able to connect with their therapist, and engage in the process, often noting that they would forget the computer screen was between them. Couples further noted that the video actually allowed for the alliance to be enhanced, as there was a greater perceived focus on the process of therapy. Furthermore couples noted feeling less judged, which in turn allowed them to disclose more vulnerable thoughts and feelings. Overall, couples found the experience to be positive and engaging, in spite of, and in many cases because of, connecting with the therapist through a computer screen.

## Data Availability Statement

The datasets generated for this study are available on request to the corresponding author.

## Ethics Statement

The studies involving human participants were reviewed and approved by Curtin University Human Research Ethics Committee. The patients/participants provided their written informed consent to participate in this study.

## Author Contributions

AK conceptualized the research and was principal author of the publication. She collected, analyzed, and interpreted the data. BB and RR were the supervisors of the study and assisted in conceptualizing and conducting the research. RK acted as an advisor in the research process. MC and MD prepared the manuscript for publication.

## Conflict of Interest

The authors declare that the research was conducted in the absence of any commercial or financial relationships that could be construed as a potential conflict of interest.
